# Effect of passive smoking exposure on risk of type 2 diabetes: a systematic review and meta-analysis of prospective cohort studies

**DOI:** 10.3389/fendo.2023.1195354

**Published:** 2023-07-31

**Authors:** Guo-Qiang Qin, Li Chen, Jun Zheng, Xiao-Min Wu, Yang Li, Kai Yang, Tong-Feng Liu, Zhong-Ze Fang, Qiang Zhang

**Affiliations:** ^1^ Department of Geriatrics, Tianjin Medical University General Hospital, Tianjin Key Laboratory of Elderly Health, Tianjin Geriatrics Institute, Tianjin, China; ^2^ Department of Toxicology and Sanitary Chemistry, School of Public Health, Tianjin Medical University, Tianjin, China; ^3^ Department of Epidemiology and Biostatistics, School of Public Health, Tianjin Medical University, Tianjin, China; ^4^ National Center for Chronic and Noncommunicable Disease Control and Prevention, Chinese Center for Disease Control and Prevention, Beijing, China; ^5^ Tianjin Key Laboratory of Environment, Nutrition and Public Health, Tianjin, China; ^6^ National Demonstration Center for Experimental Preventive Medicine Education, Tianjin Medical University, Tianjin, China

**Keywords:** passive smoking, type 2 diabetes, prospective cohort studies, meta-analysis, relative risk

## Abstract

**Objective:**

The effect of passive smoking exposure on the risk of type 2 diabetes has not been systematically studied. A meta-analysis was conducted to assess the association between passive smoking exposure and the risk of diabetes.

**Methods:**

We searched three major databases up to 31 October 2022 to identify relevant prospective cohort studies on the association between passive smoking and the risk of type 2 diabetes. The pooled relative risk (RR) and 95% confidence interval (CI) for the association between passive smoking exposure and the risk of type 2 diabetes were analyzed using a fixed-effect model.

**Results:**

Ten prospective cohort studies were included in this meta-analysis, with a total of 251,620 participants involved. The pooled RR showed a significantly positive association between nonsmokers exposed to passive smoking and type 2 diabetes as compared to non-smokers who were not exposed to passive smoking [RR = 1.27; 95% CI (1.19, 1.36); p < 0.001]. Sensitivity analysis indicated that the pooled RR was not substantially affected by any of the individual studies.

**Conclusion:**

Exposure to passive smoking increases the risk of type 2 diabetes. This study may have a positive effect on the prevention of type 2 diabetes.

**Systematic review registration:**

https://www.crd.york.ac.uk/PROSPERO/, identifier CRD42023372532.

## 1 Introduction

Diabetes is a chronic metabolic disease, and its rising prevalence is a serious public health concern globally (1). In particular, in developing countries, the prevalence of diabetes and the number of adults with the disease are increasing faster than in high-income countries (2, 3). Type 2 diabetes is the most common type of diabetes, accounting for more than 90% of patients with diabetes. As we all know, it can give rise to secondary complications such as cardiovascular disease, stroke, and diabetes retinopathy (4–6). Therefore, the identification of risk factors of type 2 diabetes is of significant importance to prevent or delay the occurrence of the disease.

Although tobacco control is a major success story in public health after the adoption of the Framework Convention on Tobacco Control, smoking remains a leading risk of premature death and disability worldwide (7). Environmental tobacco smoke contains more than 4,000 chemicals, including at least 40 carcinogens (8). In addition, in a retrospective analysis, 40% of children, 33% of male non-smokers, and 35% of female non-smokers were exposed to passive smoking worldwide (9). It has been shown that passive smoking is associated with a variety of diseases and causes a serious healthcare burden (10).

Evidence of a significant association between type 2 diabetes and active smoking has been documented (11, 12). In a meta-analysis of 25 studies, active smokers had a 44% higher risk of developing type 2 diabetes than never smokers (13). In addition, some studies have also shown that passive smoking is one of the risk factors for type 2 diabetes (14, 15). Prospective cohort studies observed that passive smoking significantly increased the risk of type 2 diabetes in never smokers, and there was a positive dose–response relationship between the passive smoking exposure level and type 2 diabetes in never smokers (16, 17). Thus, reducing exposure to tobacco smoke through effective public health and clinical interventions may avert the onset of type 2 diabetes and other related diseases.

To provide an up-to-date quantitative assessment of the association between passive smoking and type 2 diabetes, we conducted a meta-analysis based on the currently published prospective cohort studies to provide scientific and theoretical evidence for the prevention of type 2 diabetes.

## 2 Methods

The design, implementation, analysis, and reporting of our meta-analysis were reported in accordance with the PRISMA statement (**Table S1**). In addition, our study has been registered on PROSPERO (CRD42023372532).

### 2.1 Search strategy

We conducted a systematic search of PubMed, Web of Science, and Cochrane Library up to 31 October 2022 to identify relevant prospective cohort studies on the association between passive smoking and the risk of type 2 diabetes. We used the following Mesh terms and combinations of words: [“diabetes mellitus, type 2” (Mesh) OR “non-insulin dependent diabetes” OR “diabetes mellitus, type II” OR “type 2 diabetes” OR “T2DM” OR “prediabetic state” OR “glucose metabolism disorders” OR “insulin resistance” OR “hyperglycemia” OR “impaired fasting glucose”] AND [“tobacco smoke pollution” (Mesh) OR “passive smoking” OR “secondhand smoking” OR “environmental tobacco smoke pollution” OR “air tobacco smoke pollution” OR “involuntary smoking”]. The search terms used in different databases are shown in **Table S2**. The searches were limited to human studies.

### 2.2 Selection criteria and exclusion criteria

**Figure 1:**
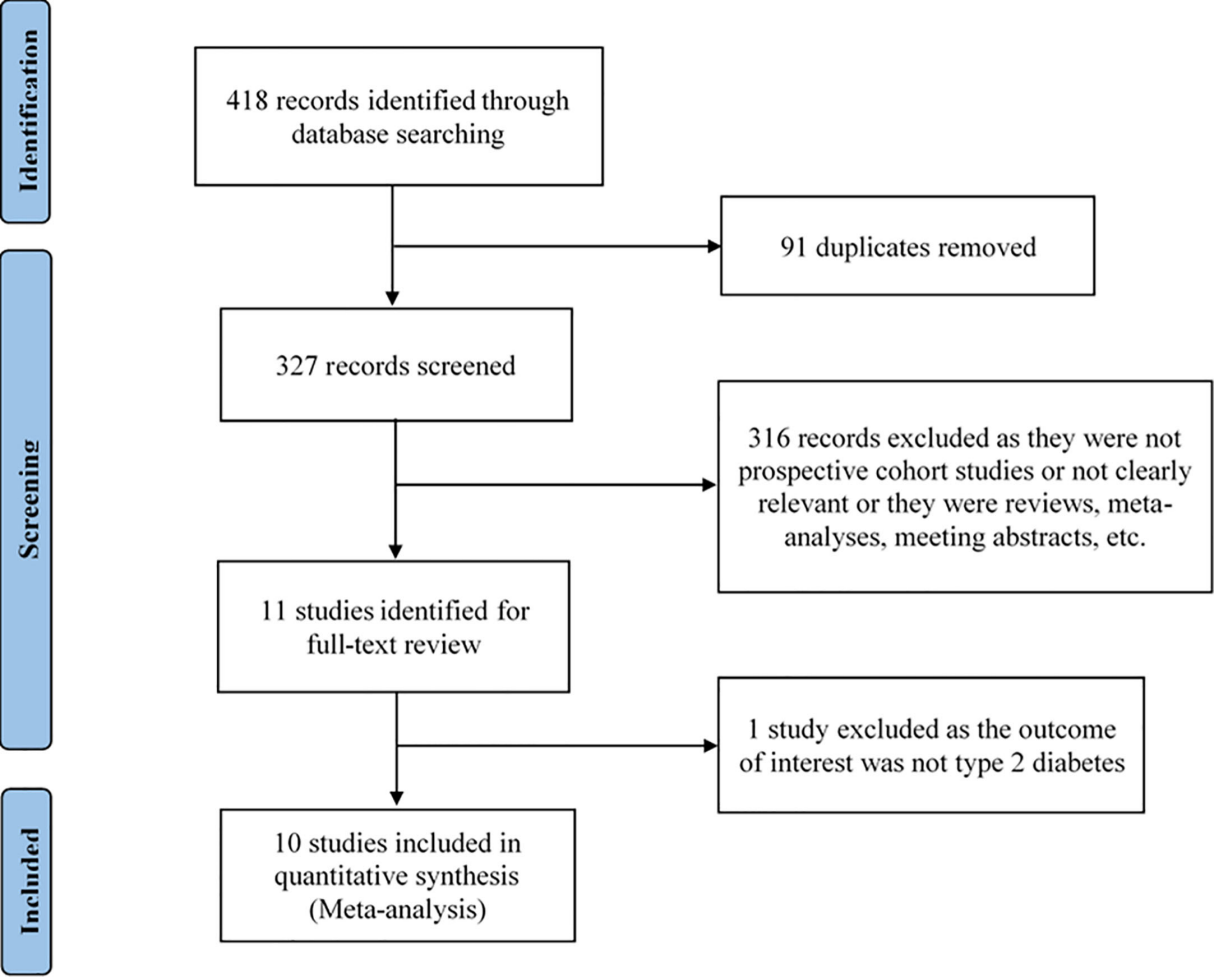
Flow chart of the selection of studies included in the meta-analysis.

We identified the possible eligible studies by performing an initial screen of identified abstracts or titles and then read the full articles to include eligible studies. The studies were selected if they met all of the following criteria: (1) being a prospective cohort study; (2) the exposure was passive smoking and the outcome was type 2 diabetes; (3) reported estimates of the relative risk (RR) or odds ratio (OR) or hazard ratio (HR) and its 95% confidence interval (CI). The studies were excluded if they met any of the following criteria: (1) being duplicate publications; (2) not being a prospective cohort study; (3) not being relevant; (4) being reviews, meta-analyses, meeting abstracts, and letters; (5) RR or OR or HR and its 95% CI not be reported. Studies with larger sample sizes were chosen among duplicate publications from the same prospective cohort study. Reviews or studies that did not report available information were excluded. The flow chart of the selection of studies is shown in **Figure 1**.

### 2.3 Data extraction and quality assessment

Two investigators independently evaluated all relevant articles and identified eligible studies from the databases. Any disagreement about whether a study met the inclusion criteria was resolved through discussion to come to an agreement. The following information of the identified studies was recorded: first author, year of publication, sample size, new cases of type 2 diabetes, follow-up year, RR or OR or HR with corresponding 95% CI, age range, percentage of female, country, define of exposed groups, exposure site, and adjusted variables for each study.

The Newcastle–Ottawa Scale (NOS) was used to assess study quality (18). This scale assigns a maximum of nine points to each study: four scores were assigned for selection of study groups, two scores were assigned for comparability of study groups, and three scores were assigned for assessment of outcomes and adequacy of follow-up. Studies with scores greater than or equal to 7 were defined as a high quality.

### 2.4 Statistical analysis

When the incidence of an outcome of interest in the study population is low, OR is close to RR (19). In addition, as HR was broadly equivalent to RR, HR was directly considered as RR (14). STATA/MP 17 for Windows was used to analyze the data. In this meta-analysis, RRs and 95% CIs were considered as the effect size for all studies. The values and corresponding standard errors of each study were converted into their natural logarithms to stabilize the variance and normalize its distribution. The fixed-effect model was used to estimate the pooled RR with corresponding 95% CI, and the inverse of the variance was weighted. The *I^2^* statistic was used to estimate the heterogeneity among the studies, and a larger value of *I^2^* indicates a greater heterogeneity, with a *p*-value < 0.10 deemed to be significant. The RRs and 95% CIs in the included studies were visualized using forest plot. A funnel plot was used to visualize potential publication bias, Egger’s linear regression test was used to measure the asymmetry of the funnel plot, and a *p*-value < 0.05 was considered significant. Sensitivity analysis was used to examine the impact of a single study. When heterogeneity is high, possible sources of heterogeneity need to be explored by subgroup analyses. All tests were two-tailed, and statistical significance was defined as *p* < 0.05.

## 3 Results

### 3.1 Study selection

The selection process of the study is shown in **Figure 1**. A total of 418 articles were identified by the search strategy. We first removed 91 articles as they were duplicates, and, then, 327 remaining articles were available for screening. By screening the titles and abstracts, 316 articles excluded as they were not prospective cohort studies or not clearly relevant or they were reviews, meta-analyses, meeting abstracts, etc. After reading the full-text of the remaining 11 articles, one article was excluded as the outcome of interest was not type 2 diabetes. Finally, we selected 10 articles meeting the inclusion criteria for data analysis.

### 3.2 Study characteristics

**Table 1** summarizes the relevant characteristics of the 10 studies included in the meta-analysis. The population size for each study ranged from 885 to 100,526, involving a total of 251,620 participants from seven countries (two studies from Korea, two studies from Japan, two studies from America, one study from China, one study from Germany, one study from France, and one study from Switzerland). Relevant details of the exposure groups and the adjusted variables for each study are shown in **Table 2**. The NOS was used to assess the quality of the included articles, and the quality score results are shown in **Table 1**. In addition, six studies were identified as of relatively high quality (≥7 points). Other characteristics of the included studies are also listed in **Table 1**.

**Table 1:** Characteristics of the studies included in the meta-analysis.

First Author	Year	Country	Sample Size	No. of New Type 2 Diabetes Case	Follow-up Year	Age Range	%Female	Quality Score
Hayashino Y (20)	2008	Japan	6,498	229	3.4	19–69	20.90%	6
Kowall B (21)	2010	Germany	885	93	7	55–74	44%	8
Ko KP (17)	2011	Korea	4,442	465	6	40–69	84%	6
Zhang L (22)	2011	America	100,526	5,392	24	41–55	100%	7
Lajous M (23)	2013	France	37,343	795	13.4	NA	100%	8
Eze IC (16)	2014	Switzerland	6,392	315	11	29–73	5%	7
Jeon J (24)	2019	Korea	2,079	200	11.6	≥40	86.50%	6
Jiang LH (25)	2019	America	39,887	2,495	17	NA	100%	8
Huang C (26)	2020	China	28,177	774	7.3	30–79	100%	7
Oba S (27)	2020	Japan	25,391	708	10	40–69	100%	6

NA, not recorded or available.

**Table 2:** Relevant details for each study.

First Author	Year	Define of Exposed Groups	Exposure Site	Adjusted Variables
Hayashino Y (20)	2008	Those currently exposed to passive smoke but who did not actively smoke, irrespective of past smoking	Workplace	Age, sex, BMI, physical activity, alcohol, family history of diabetes, hypertension, health promotion intervention, frequency of sweetened beverage and vegetable intake, do not care about eating too much fat at all
Kowall B (21)	2010	Never smokers exposed to passive smoke	Total	Age, sex, parental diabetes, socioeconomic status, alcohol intake, physical activity, waist circumference, blood pressure, hypertriglyceridemia, HDL cholesterol, log insulin, log adiponectin, intake of meat, sausage, salad, vegetables, whole-grain bread, coffee consumption
Ko KP (17)	2011	Never smokers exposed to passive smoke	Total	Age, sex, residential area, education level, alcohol consumption, waist circumference, regular exercise, hypertension history, total cholesterol, and HOMA-IR, baseline glucose tolerance status
Zhang L (22)	2011	Never smokers regularly exposed to passive smoke	Total	Age, race, BMI, physical activity, husband’s education, family history of diabetes, total energy intake, intake of alcohol, magnesium, calcium, vitamin D, total trans-fat, fiber from cereal, caffeine, total fat, saturated fat
Lajous M (23)	2013	Never smokers exposed to passive smoke ≥ 4 h/day	Total	Age, BMI, parental history of diabetes, education, body silhouette at age 8, childhood secondhand smoke exposure, physical activity, menopause, hormone replacement therapy, treated hypercholesterolemia and hypertension, alcohol, coffee, processed red meat consumption
Eze IC (16)	2014	Never smokers exposed to passive smoke	Total	Age, sex, BMI, educational attainment, area-level socio-economic position, alcohol consumption, smoking pack-years, work exposure to dust gas and fumes, citrus fruits, other fruits and raw vegetables, physical activity, home outdoor PM10
Jeon J (24)	2019	The “high-to-low” trajectory of passive smoke exposure levels by never smokers	Total	Age, sex, family history of type 2 diabetes, alcohol drinking status, physical exercise, BMI, systolic blood pressure, total cholesterol level, educational levels, household income at baseline, total cholesterol level at end line
Jiang LH (25)	2019	Both childhood and adult exposed to passive smoke but who never smoke	Household	Age, BMI, race, family history of diabetes, physical activity, alcohol consumption, dietary factors (daily dietary calories, magnesium, calcium, vitamin D, dietary fiber, total fat, saturated fat)
Huang C (26)	2020	Never smokers exposed to passive smoke	Total	Age, BMI, marital status, level of education, annual household income, alcohol consumption, intake frequency of red meat, vegetables and fruit, physical activity, family history of diabetes, menopause status, use of oral contraceptives, hypertension
Oba S (27)	2020	Never-smoking women whose husband smoked ≥ 40 cigarettes/day, and exposed to passive smoke	Household	Age, nine regions, the term of survey, BMI, hypertension, parental history of diabetes, leisure-time physical activity, intakes of coffee and alcohol, workplace (or public facilities) passive smoke exposure

BMI, body mass index; HDL, high-density lipoprotein; HOMA-IR, homeostasis model assessment of insulin resistance; PM10, particulate matter < 10 μm in diameter.

### 3.3 Effect of passive smoking exposure on risk of type 2 diabetes

**Figure 2:**
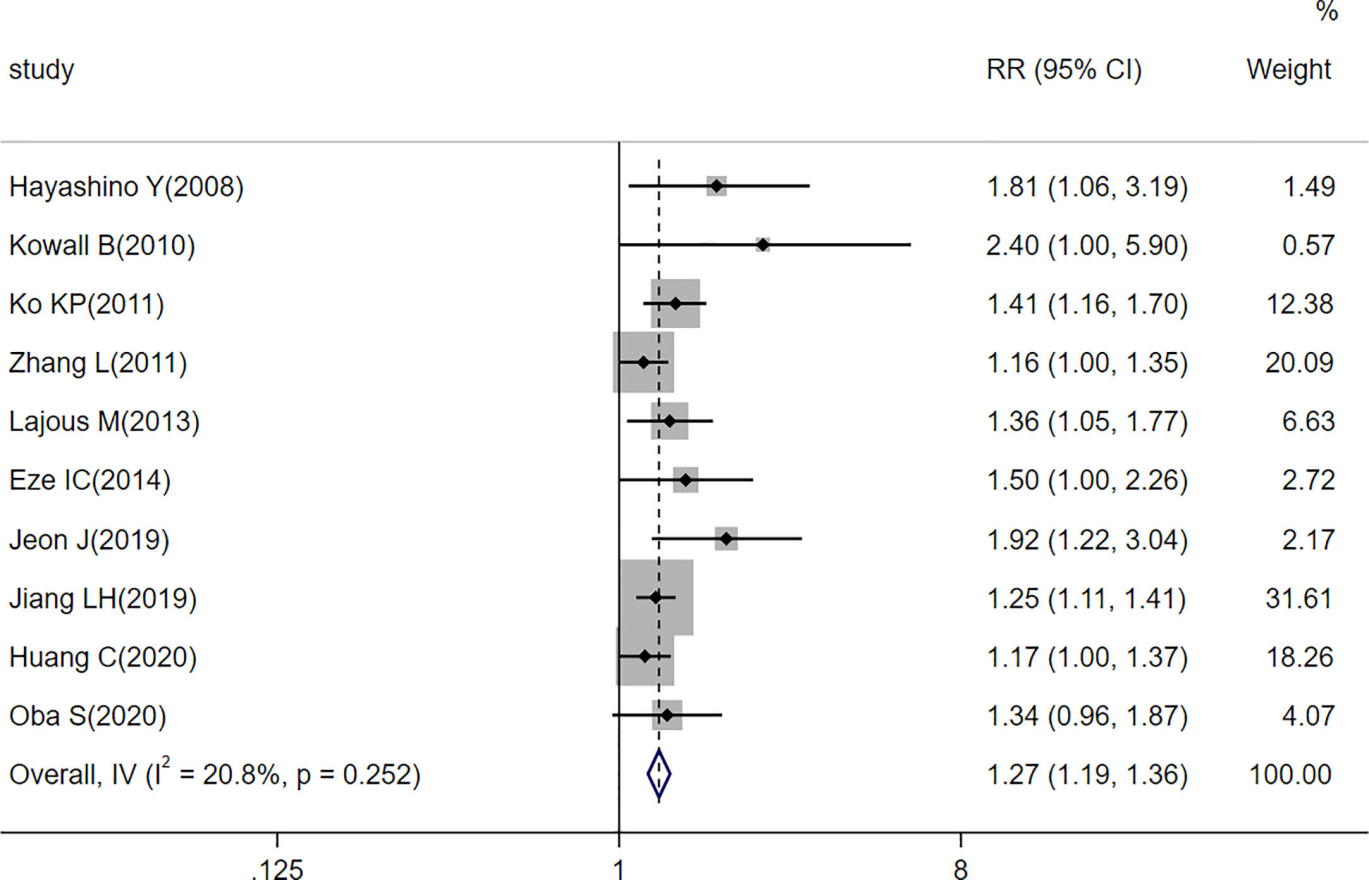
Forest plot of associations between passive smoking and type 2 diabetes risk.

**Figure 3:**
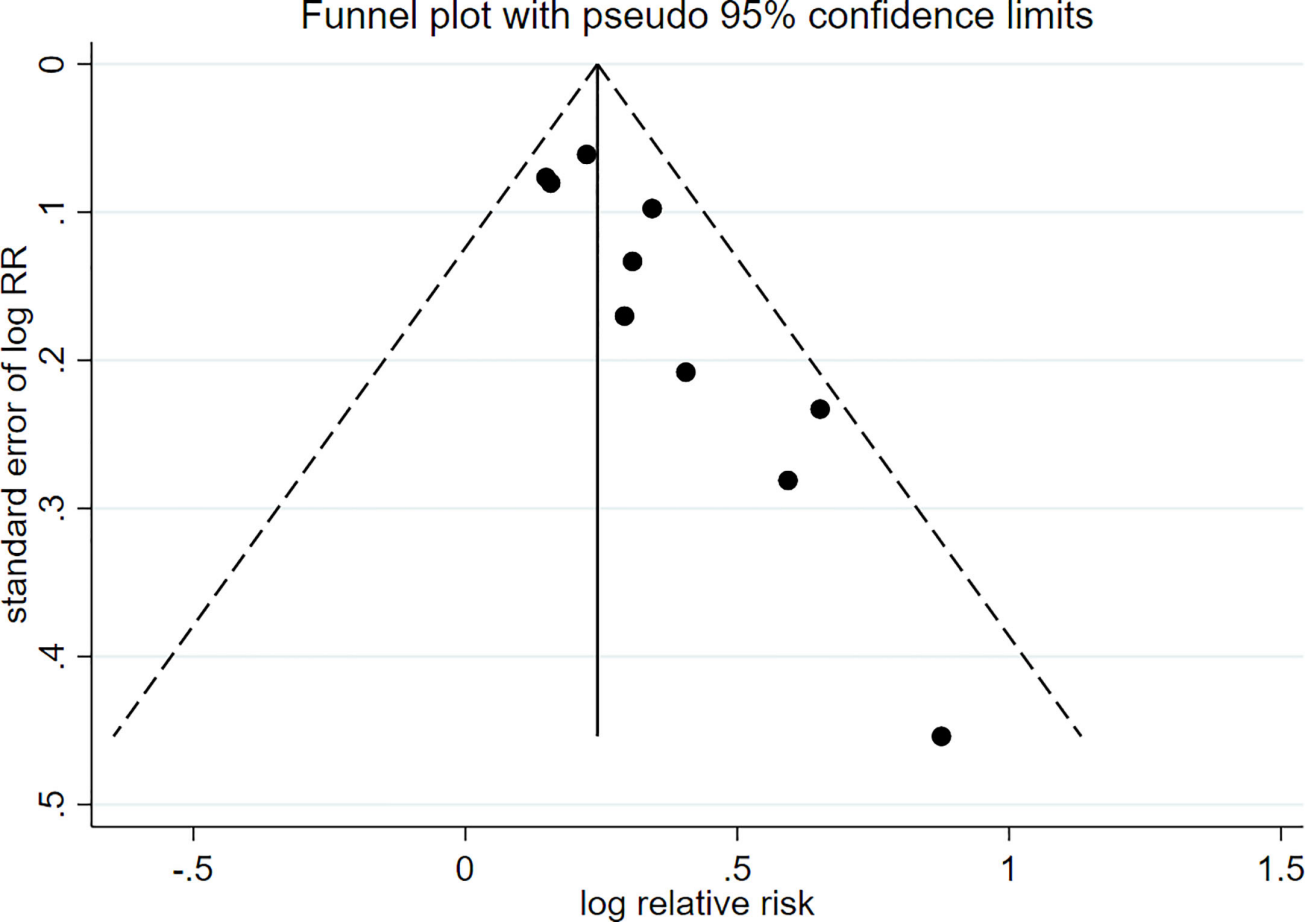
Funnel plots for identifying publication bias in the meta-analysis.

There were 10 prospective cohort studies evaluating the risk of passive smoking with type 2 diabetes. **Figure 2** shows a forest plot of the pooled analysis results (pooled RR, 1.27; 95% CI, 1.19–1.36). Compared with non-smokers without passive smoking exposure, non-smokers exposed to passive smoking have an increased risk of type 2 diabetes. There was low heterogeneity between studies (*p* = 0.252, *I^2 = ^* 20.8%). The funnel plot in **Figure 3** indicates that publication bias was found in the meta-analysis (*p* < 0.05, Egger’s test).

### 3.4 Sensitivity analysis

Sensitivity analysis examined the effect on the pooled RR by deleting one study each time. **Table 3** shows that passive smoking was significantly associated with a greater risk of type 2 diabetes compared with non-exposed group after deleting individual studies one by one, indicating that the pooled RR was not substantially affected by any of the individual studies.

**Table 3:** RR estimates and 95% CI after omitting studies one by one.

Study Omitted	RR	95% CI
Hayashino Y (2008) (20)	1.27	(1.19, 1.36)
Kowall B (2010) (21)	1.27	(1.19, 1.36)
Ko KP (2011) (17)	1.26	(1.17, 1.35)
Zhang L (2011) (22)	1.31	(1.21, 1.41)
Lajous M (2013) (23)	1.27	(1.18, 1.36)
Eze IC (2014) (16)	1.27	(1.19, 1.36)
Jeon J (2019) (24)	1.26	(1.18, 1.35)
Jiang LH (2019) (25)	1.29	(1.19, 1.40)
Huang C (2020) (26)	1.30	(1.21, 1.40)
Oba S (2020) (27)	1.27	(1.19, 1.36)
Combined	1.27	(1.19, 1.36)

RR, relative risk; CI, confidence interval.

## 4 Discussion

Previous epidemiological studies have found that passive smoking is associated with the risk of type 2 diabetes (26, 27). On the basis of the data from 10 prospective cohort studies, this meta-analysis found that exposure to passive smoking increased the risk of type 2 diabetes in non-smokers compared to non-smokers who were not exposed to passive smoking [overall RR = 1.27; 95% CI (1.19, 1.36); *p* < 0.001].

Type 2 diabetes is influenced by a variety of environmental factors, among which tobacco exposure may be an important risk factor (28). Active smoking has been widely established as a risk factor for type 2 diabetes, although there are relatively few studies on the association between passive smoking and type 2 diabetes (21). The main component of environmental tobacco smoke is sidestream smoke, whose emissions are generally larger than those of mainstream smoke (8). Many toxic components are found in higher concentrations in sidestream smoke than those directly inhaled from cigarettes, such as about five times as much carbon monoxide, about three times as much cigarette tar in sidestream smoke as smoke inhaled directly from cigarettes (29, 30). Compared with active smoking, the negative effects of passive smoking are more widespread, especially for children and adolescents (31). We cannot ignore the fact that passive smoking can occur in many sites, such as household, public places, and even in the workplace. In addition, some studies have found a positive dose–response relationship between the level of environmental tobacco smoke exposure and type 2 diabetes in non-smokers (16, 25). Thus, reducing exposure to passive smoking may have a positive effect on the prevention of type 2 diabetes.

Our findings suggest that passive smoking is associated with type 2 diabetes, which is consistent with other research findings (14, 15). This association may involve several mechanisms: (1) Environmental tobacco smoke contains a large number of toxic substances, among which nicotine is an important ingredient. Nicotine can develop insulin resistance by negatively affecting insulin action (32). In addition, nicotine was found to induce β cell damage through direct interaction with acetylcholine receptors, resulting in reduced insulin production (33, 34). (2) Tobacco smoke exposure decreases insulin secretion by reducing antioxidant production and inducing pancreatic β cells sensitivity to reactive oxygen species, thereby promoting the occurrence of type 2 diabetes (35, 36). (3) Environmental tobacco smoke contains at least 40 carcinogens, and studies have shown that tobacco smoke may cause pancreatic cancer by inducing chronic inflammatory responses (37). This suggests that carcinogens in tobacco smoke have a chronic toxic effect on the pancreas, which, in turn, affects glucose metabolism.

In recent years, environmental pollution caused by passive smoking has received more attention. Passive smoking not only affects the occurrence of type 2 diabetes but also is associated with respiratory diseases, cardiovascular diseases, and cancer (38, 39). In addition, the existence of diabetes will also lead to a series of secondary diseases and affect all organ systems in the body, including diabetic retinopathy, diabetic nephropathy, and diabetic neuropathy (40–42). The hazards of passive smoking are complex, and we should try to avoid tobacco smoke exposure to achieve the purpose of preventing type 2 diabetes.

There are several potential limitations in this study. First, our meta-analysis included only the literature published in English, and, thus, the actual total number of eligible studies may be larger than the number of studies that we included. Second, because the exposure status of passive smoking is self-reported, there may be inaccurate reporting by participants that influence the risk estimates. Finally, on the basis of the fact that the research studies that we included are observational studies, they cannot clarify the causation. Furthermore, there was publication bias in our meta-analysis, whose main source was due to negative results that are not reported.

## 5 Conclusions

In conclusion, our study suggests that exposure to passive smoking increases the risk of developing type 2 diabetes. The finding has important public health implications for guiding the prevention of type 2 diabetes. By taking appropriate measures to reduce indoor tobacco smoke exposure, the risk of type 2 diabetes may be decreased. An additional meta-analysis that includes well-designed cohort or intervention studies is needed to confirm the current findings.

## Data availability statement

The original contributions presented in the study are included in the article/**Supplementary Material**. Further inquiries can be directed to the corresponding author.

## Author contributions

Conception and design: QZ and Z-ZF. Search strategy, data extraction, analysis, and writing: G-QQ and LC. Statistical expertise and corrections: JZ, X-MW, YL, KY, and T-FL. All authors contributed to the article and approved the submitted version.
