# Supplementary material for: Effect of passive smoking exposure on risk of type 2 diabetes: a systematic review and meta-analysis of prospective cohort studies
Source: Front Endocrinol (Lausanne). 2023 Jul 31;14:1195354. doi: 10.3389/fendo.2023.1195354 (PMC10432686; doi:10.3389/fendo.2023.1195354)
Supplement: Supplementary file 1 [file Table_1.docx]

**Table S1** The PRISMA checklist

| **Section and Topic** | **Item** | **Location where item is reported** |
| --- | --- | --- |
| **TITLE** | | |
| Title | 1 | Identify the report as a systematic review and a meta-analysis. |
| **ABSTRACT** | | |
| Abstract | 2 | The structured abstract includes Objective, Methods, Results and Conclusion. |
| **INTRODUCTION** | | |
| Rationale | 3 | Described in the Introduction. |
| Objectives | 4 | Described in the Abstract and the Introduction. |
| **METHODS** | | |
| Eligibility criteria | 5 | Defined in the Methods. |
| Information sources | 6 | Described in the Methods. |
| Search strategy | 7 | Described in the Methods. |
| Selection process | 8 | Described in the Methods. |
| Data collection process | 9 | Described in the Methods. |
| Data items | 10 | Described in the Methods and summarized in Table 1 and Table 2. |
| Study risk of bias assessment | 11 | Assessed with Newcastle-Ottawa scale and described in the Methods. Shown in Table 1 and Appendix Table s2. |
| Effect measures | 12 | Relative Risk |
| Synthesis methods | 13 | Described in Statistical analysis and reported in detail in Results. |
| Reporting bias assessment | 14 | This have not provided. |
| Certainty assessment | 15 | This have not provided. |
| **RESULTS** | | |
| Study selection | 16 | See Flow Diagram in Figure 1. |
| Study characteristics | 17 | Described in Table 1 and Table 2. |
| Risk of bias in studies | 18 | Assessed with Newcastle-Ottawa scale and described in Table 1 and Appendix Table s2. |
| Results of individual studies | 19 | Described in Results and shown in Figure 2 and Table 2. |
| Results of syntheses | 20 | Described in Results and shown in Figure 2 |
| Reporting biases | 21 | Described in the Discussion. |
| Certainty of evidence | 22 | This have not provided. |
| **DISCUSSION** | | |
| Discussion | 23 | All details described in the Discussion. |
| **OTHER INFORMATION** | | |
| Registration and protocol | 24 | The protocol is described in the Methods. The meta-analysis has registered in  PROSPERO website (https://www.crd.york.ac.uk/prospero/) |
| Support | 25 | Describe sources of financial or non-financial support for the review, and the role of the funders or sponsors in the review. |
| Competing interests | 26 | None. |
| Availability of data, code and other materials | 27 | The data that support the findings of this study are available on request from  the corresponding authors. |

**Table S2** Search terms used by different databases

| **Database** | **Search terms or combinations of the search terms** |
| --- | --- |
| PubMed | (“diabetes mellitus, type 2” [Mesh] OR “non-insulin dependent diabetes” OR “diabetes mellitus, type II” OR “type 2 diabetes” OR “T2DM” OR “prediabetic state” OR “glucose metabolism disorders” OR “insulin resistance” OR “hyperglycemia” OR “impaired fasting glucose”) AND (“tobacco smoke pollution” [Mesh] OR “passive smoking” OR “secondhand smoking” OR “environmental tobacco smoke pollution” OR “air tobacco smoke pollution” OR “involuntary smoking”) |
| Web of Science | (“diabetes mellitus, type 2” OR “non-insulin dependent diabetes” OR “diabetes mellitus, type II” OR “type 2 diabetes” OR “T2DM” OR “prediabetic state” OR “glucose metabolism disorders” OR “insulin resistance” OR “hyperglycemia” OR “impaired fasting glucose”) AND (“tobacco smoke pollution” OR “passive smoking” OR “secondhand smoking” OR “environmental tobacco smoke pollution” OR “air tobacco smoke pollution” OR “involuntary smoking”) |
| Cochrane Library | (“diabetes mellitus, type 2” OR “non-insulin dependent diabetes” OR “diabetes mellitus, type II” OR “type 2 diabetes” OR “T2DM” OR “prediabetic state” OR “glucose metabolism disorders” OR “insulin resistance” OR “hyperglycemia” OR “impaired fasting glucose”) AND (“tobacco smoke pollution” OR “passive smoking” OR “secondhand smoking” OR “environmental tobacco smoke pollution” OR “air tobacco smoke pollution” OR “involuntary smoking”) |

**Table S3** Study quality scores included in the meta-analysis

| First author | Selection | | | |  | Comparability |  | Outcome | | |
| --- | --- | --- | --- | --- | --- | --- | --- | --- | --- | --- |
|  | Whether the exposed cohort is representative | Selection of the non-exposed cohort | Identification of exposure factor | There is no outcome event before the study begins |  | Comparability of cohorts on the basis of the design or analysis |  | Evaluation of outcome event | Whether the follow-up was long enough to produce outcome event | adequacy of follow-up of cohorts |
| Hayashino Y | 0 | * | * | * |  | * * |  | * | 0 | 0 |
| Kowall B | * | 0 | * | * |  | * * |  | * | * | * |
| Ko KP | * | * | 0 | * |  | * |  | * | * | 0 |
| Zhang L | 0 | * | 0 | * |  | * * |  | * | * | * |
| Lajous M | * | * | 0 | * |  | * * |  | * | * | * |
| Eze IC | * | 0 | * | * |  | * * |  | * | * | 0 |
| Jeon J | * | 0 | 0 | * |  | * * |  | * | * | 0 |
| Jiang LH | * | * | 0 | * |  | * * |  | * | * | * |
| Huang C | 0 | * | 0 | * |  | * * |  | * | * | * |
| Oba S | 0 | * | 0 | * |  | * * |  | * | * | 0 |
